# The Black Cloud of Endometriosis on different aspects of the Life of Women with Endometriosis: a qualitative study in Iran

**DOI:** 10.1186/s12888-024-06405-8

**Published:** 2024-12-27

**Authors:** Sanaz Mollazadeh, Mojgan Mirghafourvand, Khadijeh Mirzaii Najmabadi, Talat Khadivzadeh

**Affiliations:** 1 Department of Midwifery, School of Nursing and Midwifery, Mashhad University of Medical Sciences, Mashhad, Iran; 2 Social Determinants of Health Research Center, Tabriz University of Medical Sciences, Tabriz, Iran; 3 Nursing and Midwifery Care Research Center, Mashhad University of Medical Sciences, Mashhad, Iran

**Keywords:** Endometriosis, Perception, Qualitative research, Quality of life, Women’s lives

## Abstract

**Aim:**

To explore the perception of the impact of endometriosis on various aspects in affected women with endometriosis.

**Method:**

Data was gathered through in-depth and semi-structured individual interviews using open questions to comprehend women’s experiences with endometriosis and their health-promoting lifestyle. The Conventional Qualitative Content Analysis approach was utilized, and sampling continued until data saturation. Ultimately, 14 women with endometriosis were interviewed.

**Results:**

The theme was following the dominance of the Black Cloud of Endometriosis on Different Aspects of the Life of Women with Endometriosis; with main categories involving (a) Physical depreciation caused by endometriosis, (b) psychological deterioration caused by endometriosis, (c) social isolation, (d) marital relationship disorder, and sexual dissatisfaction.

**Conclusions:**

The concepts show that endometriosis impacts negatively on different aspects of women’s lives. Considering that endometriosis is a multi-dimensional disease, identifying the different aspects of this disease and paying attention to meeting the perceived needs of the affected women will help to improve the treatment process and bring the affected women to the end of their health. These should be improved by adopting appropriate approaches in health policies.

**Supplementary Information:**

The online version contains supplementary material available at 10.1186/s12888-024-06405-8.

## Background

Endometriosis is a prolonged, benign disorder in women, it is defined as the presence of endometrial tissue outside the uterine, with a prevalence of 2–10% in women of reproductive age [1]. Endometriosis commonly presents as lesions or cysts in various areas of the body, including the peritoneum and pelvic organs, impacting both physical and psychological well-being and overall quality of life [2]. Endometriosis may remain asymptomatic in certain patients and be accidentally discovered [3]. It is associated with several symptoms that can be disturbing for patients and affect their quality of life and ranks as the third most common reason for hospitalization in the US, often leading to high rates of hysterectomy [4]. There is no definitive cure for endometriosis, and women who suffer from this chronic, estrogen-dependent disease may experience a range of symptoms, from mild pain to very debilitating disease [5]. Studies have shown no link between endometriosis severity and disease stage and symptom severity [6–9]. Common symptoms of this disease include dyspareunia, infertility, dysmenorrhea, and dyschezia. Endometriosis has no definitive cure, and women affected by this chronic, estrogen-dependent condition may range from experiencing mild pain to severe disability [5]. The etiology of endometriosis is complex and multifactorial [10]. Although the precise cause of endometriosis remains unclear, retrograde menstruation is widely acknowledged as a major contributing factor [11, 12]. Numerous studies in Iran and worldwide indicate that endometriosis significantly impacts women’s lives [13]. This condition can affect various aspects including physical, psycho-emotional, and social dimensions, lifestyle, financial status, employment, and education, as well as important life milestones like marriage and parenthood. In essence, women’s entire lives can be negatively influenced by contracting endometriosis and its consequences [5, 14, 15]. Daily routines, social harmony, interpersonal relationships, and overall health can all be disrupted [16, 17]. The results of qualitative studies that have investigated the impact of endometriosis on women’s lives have shown extensive effects in different dimensions, such as physical health, psychological well-being, social interactions, sexual and marital relationships, economic factors, employment and occupational dimensions, education, and lifestyle [5, 14, 18]. Considering that endometriosis is a chronic condition with no definitive cure that greatly impacts women’s lives [19], it is important to explore the issues and outcomes of endometriosis to gather valuable information that can enhance the quality of life for affected women. Regrettably, endometriosis is a highly distressing ordeal for many women, yet their issues often go undiagnosed, resulting in the long-term repercussions of the condition being disregarded and leading to prolonged suffering [20]. Unfortunately, endometriosis is a profoundly distressing experience and women’s problems are underdiagnosed, so the lasting consequences of the situation are ignored and lead to long-term distress [20]. This disease, like diabetes and Crohn’s disease, imposes a significant economic burden on individuals and society [14]. Therefore, adopting effective health care appears as a useful and necessary approach for women with endometriosis [1, 3, 21]. In the present study, we intend to explore the impact of endometriosis on various aspects of women’s lives.

### Study aim

To explore the perception of the impact of endometriosis on various aspects of women’s lives.

## Method and materials

### Study type and participants

This article was extracted from the qualitative segment of mixed-method research employing a sequential explanatory design. The study was conducted from March 2023 to August 2023 in Iran, with qualitative participants comprising extreme cases [22], Participants who achieved 10% of the upper and lower limits in overall health-promoting lifestyle were chosen in the quantitative analysis. The results of this study were reported using the COREQ^1^ checklist [23].

### Eligibility criteria

The study included women aged 15 to 49 years diagnosed with endometriosis through open surgery, laparoscopy, histological diagnosis, presence of endometrioma cyst, or confirmed by ultrasound/MRI. Participants had localized pelvic/peritoneal endometriosis, experienced symptoms for at least one year, had no endometriosis in other organs, were Iranian, married, able to answer questions, non-menopausal (no amenorrhea for over a year), and did not have major diseases like mental disorders, severe depression, diabetes, cancer, etc.

### Sampling

Participants were carefully selected to ensure a wide range of age, education, occupation, disease awareness duration, reproductive status, disease severity, and symptoms. Selection criteria included individual statements and high or low scores in the quantitative study. Eligible women were briefed on the research objectives and written informed consent was obtained from those willing to participate. Interviews took place in health centers selected by the participants, and sampling continued until data saturation, indicating no new information or code was obtained [24]. In this study, data repetition started at the 8th interview, with 14 participants at data saturation. Interviews averaged 60 min (range 60 to 120 min).

### Data collection method

Data collection began after the thesis proposal was approved at Mashhad Nursing and Midwifery College. The design code and ethics approval were obtained from the Ethics Committee of Mashhad University of Medical Sciences (ethics code: IR.MUMS.NURSE.REC. 1401. 064). This study originates from the qualitative component of the PhD thesis titled “Designing and Validating a Health Promotion Program for Women with Endometriosis,” which utilized a mixed-methods sequential explanatory design. This comprehensive approach involved collecting, analyzing, and integrating both quantitative and qualitative data, guided by the principles of the pragmatism paradigm. In this study, quantitative data were gathered in the first phase on 200 women with endometriosis, based on the results of the average score of the overall health-promoting lifestyle and the effects of the endometriosis disease that were obtained in the quantitative section, participants in the qualitative part consisted of extreme cases [22], that is, those who gained 10% of the upper and lower limits of the overall health-promoting lifestyle, and both data types were integrated to develop the health promotion program. Data collection occurred through in-depth and semi-structured individual interviews with open-ended questions. The interview guide was prepared before the qualitative phase, ensuring data validity and alignment with research objectives. The interviews started with preset questions, progressing to more detailed inquiries. Initially, participants were asked to provide a full introduction, answering questions like “Please share your experience with endometriosis. How did you first learn about your condition?” Subsequent questions delved into participants’ experiences with endometriosis and methods for maintaining health. Probing questions such as “Can you elaborate further?” and “What is the significance of this?” were used to garner detailed responses. The interviews concluded with open-ended prompts and an invitation for additional insights. After discussing the possibility of follow-up interviews and sharing researcher contact details, the sessions ended. Participant consent was obtained for the audio recording of the interviews, which were transcribed verbatim post-session and augmented with observational notes for comprehensive data collection. The interviews continued until data saturation was achieved.

### Data analysis

Based on the recommended stages of Graneheim & Lundman [25], Conventional content analysis was used in this study for data analysis. The analysis began by reading the entire text multiple times to fully comprehend it. Next, each word was carefully examined to identify codes. Initially, keywords reflecting the main concepts were identified. Labels encompassing multiple main ideas were directly extracted during this phase. Subsequently, the codes were organized according to their interrelations or distinctions. Data analysis was conducted alongside data collection using MAXQDA 20 software.

### Trustworthiness

Four criteria for evaluating the accuracy of qualitative studies have been proposed: Credibility, Consistency or Dependability, and Conformability. Among the various criteria for assessing the scientific rigor of qualitative research, those introduced by Goba and Lincoln stand out as particularly comprehensive [26]. In this study, advisors and counselors assisted researchers in verifying data accuracy through peer checks. Additionally, three participants reviewed coded texts to ensure correctness, suggest corrections, and highlight similar experiences (member checks). Dependability is vital in qualitative content analysis, from data collection to reporting. To enhance reliability, the researcher meticulously documented decisions and analysis activities, including primary codes, examples of theme extraction, and interview excerpts. Various measures were implemented to improve confirmability, such as excluding the researcher’s opinions, publishing results in a journal, employing design arbitration for both dependability and confirmability, and establishing decision-making rules for data interpretation. To ensure transferability, the study comprehensively described the research process and participants, allowing readers to evaluate the generalizability of the findings.

## Results

After 14 interviews with women with endometriosis (Tables 1 and 2), a total of 151 initial codes in a theme, 4 main categories, and 29 sub-categories were extracted (Table 3).

**Table 1:** Sociodemographic characteristics of women with endometriosis (*n* = 14)

Characteristic	Number (Percent)
Age (Year)	37.7 (7.3)*
Level of Education	
Elementary/Secondary school	2 (14.3)
High School/Diploma	3 (21.4)
Academic	9 (64.3)
Job	
Housewife	7 (50.0)
Employed	7 (50.0)
Adequacy of monthly income	
Less than living expenses	4 (28.6)
Equal of living expenses	6 (42.9)
More than living expenses	4 (28.6)

*Mean ± SD

**Table 2:** Demographic characteristics of the participants of the qualitative section

Row	Age (years)	Education	Occupation re	Duration of illness	Fertility status	Degree of illness	Severity of illness	Duration of interview	Score of the quantitative phase
1	41	diploma	Housekeeper	14 years	Having a child and secondary infertility	Stage IV	Long-term severe symptoms without diagnosis	an hour	high score
2	43	Bachelor of Environmental Health	Employee of the relief committee	2 years	Having a child/ starting symptoms after the birth of the child and not wanting to have a second pregnancy	Stage III	Long-term severe symptoms without diagnosis	two hours	high score
3	29	dentist	dentist	2 years	Egg freezing candidate	Stage IV	Long-term severe symptoms without diagnosis	an hour	high score
4	25	Bachelor of Educational Sciences	the teacher	10 years	Primary infertility	Stage IV	Without symptoms until emergency surgery and encountering a half kilo endometrioma cyst	an hour	high score
5	47	Master of Educational Psychology	Housekeeper	3 years	Having two children/ the onset of symptoms after the birth of children	Stage IV	Asymptomatic to heavy bleeding with clots in the last two years	an hour	high score
6	32	Bachelor of Midwifery	Housekeeper	10 years	Having a child and trying to get pregnant 6 years after endometriosis cyst surgery and getting pregnant within 6 months after trying to get pregnant	Stage IV	Long-term severe symptoms without diagnosis	an hour	low score
7	34	diploma	Housekeeper	9 years	Having three children and a third pregnancy at the same time as taking pills to treat endometriosis after surgery	Stage IV	Mild symptoms	an hour	low score
8	47	Bachelor of Midwifery	midwife	5 years	Primary infertility	Stage III	No symptoms to mild symptoms to 5 cm endometrioma cyst surgery	an hour and a half	low score
9	48	diploma	Housekeeper	12 years	Having a child and secondary infertility	Stage IV	Long-term severe symptoms without diagnosis	an hour and a half	high score
10	38	PhD in architecture	the architect	10 years	Primary infertility	Stage IV	Moderate symptoms until encountering a large mass on ultrasound	an hour and a half	high score
11	36	elementary	Housekeeper	7 years	having three children/ the onset of symptoms after the second child and the third pregnancy with a gap of 7 years from the second pregnancy, within 6 months after trying to get pregnant without endometriosis cyst surgery	Stage III	Mild symptoms	an hour and a half	low score
12	36	diploma	Insurance marketer	11 years	Having one child and secondary infertility	Stage IV	Asymptomatic until referral for secondary infertility	an hour	low score
13	32	Senior Nano	Housekeeper	10 years	Primary infertility	Stage IV	severe symptoms	an hour	low score
14	46	Bachelor of Laws	Housekeeper	8 years	Having two children/second pregnancy following the treatment of secondary infertility due to the presence of adhesions	Stage III	No sign	an hour and a half	high score

**Table 3:** Themes, main categories, and subcategories

Theme	Main category	Subcategory
The Dominance of the Black Cloud of Endometriosis on Different Aspects of the Life of Women with Endometriosis	Physical depreciation caused by endometriosis	severe menstrual pain
		heavy menstrual bleeding
		increased pain post-cesarean surgery
		presence of endometriosis cysts
		gastrointestinal complications
		reliance on continuous medication
		physical limitations in managing daily life
	Psychological deterioration caused by endometriosis	psychological distress
		worry about passing the disease on to female offspring
		worry about the future
		worry about the patient’s family member
		worry about not getting pregnant
		facing the intense stress of the illness
		lack of support from spouse and family member
		feelings of isolation in times of suffering and trying to fill the emotional gaps
		frustration with the behavior of others
		concealing the illness from others to avoid added stress
		loss of hope for normalcy
		feeling trapped in suffering until death
		escaping painful memories at the peak of illness
		continuous pain and suffering
		positive or negative challenges in dealing with others
		fatigue from being ill
		ineffectiveness of infertility treatments, frustration, and tensions caused by infertility
	Social isolation	endometriosis as a hindrance to career progression
		endometriosis as an obstacle to engaging in social activities
	Marital relationship disorder, and sexual dissatisfaction	concealing the physical, psychological, and financial challenges of endometriosis from the spouse
		marital and sexual problems
		feelings of inferiority and loss of femininity

### The dominance of the Black Cloud of Endometriosis on different aspects of the Life of Women with Endometriosis

The dominance of the black cloud of endometriosis on different aspects of the life of women with endometriosis is the concept that shows the negative effects of endometriosis on different aspects of the life of women with endometriosis. This concept emerged, with main categories involving (a) Physical depreciation caused by endometriosis, (b) psychological deterioration caused by endometriosis, (c) social isolation, (d) marital relationship disorder, and sexual dissatisfaction.

#### Physical depreciation caused by endometriosis

This main category outlines the physical discomfort experienced by women affected by endometriosis. The sub-categories include (a) severe menstrual pain, (b) heavy menstrual bleeding, (c) increased pain post-cesarean surgery, (d) presence of endometriosis cysts, (e) gastrointestinal complications, and (f) reliance on continuous medication (g) physical limitations in managing daily life.

##### Severe menstrual pain

[I started menstruating at eleven, entering puberty early. My periods were consistently painful, disrupting my daily life. (Participant 13, from lower limit)]

##### Heavy menstrual bleeding

[Since my first period, I experienced severe pain and heavy bleeding with clots, akin to a faucet turning on when I stood up. (Participant 2, from upper limit)]

##### Increased pain post-cesarean surgery

[Post-cesarean section, the pain was so intense that even morphine couldn’t alleviate it. (Participant 5, from upper limit)]

##### Presence of endometriosis cysts

[Diagnosed with a dangerous ovarian cyst, I underwent surgery where they removed my left ovary. During the procedure, an endometrioma cyst ruptured, spreading in my abdomen. (Participant 10, from upper limit)]

##### Gastrointestinal complications

[Facing bowel surgery, intestinal adhesions, and obstruction, 12 centimeters of intestine were removed, revealing endometriosis per pathology results. Despite recovery, I continued to suffer from pain, bleeding, constipation, and bloating. (Participant 10, from upper limit)]

##### Reliance on continuous medication

[Though hormonal drugs cause anxiety, I must take them indefinitely. They also lead to significant weight gain. (Participant 8, from upper limit)]

##### Physical limitations in managing daily life/ g) severe menstrual pain

[These pains rendered me unable to work, relying on my sister and mother for household chores. Endometriosis completely upended my life. (Participant 4, from lower limit)]

In general, due to the wear and tear caused by endometriosis, the dominance of this condition casts a shadow on the lives of affected women, like a black cloud or a ghost, with painful symptoms. The annoying and long-term side effects have overshadowed various aspects of their lives and disrupted the process of living a normal life.

#### Psychological deterioration caused by endometriosis

This concept explores the psychological and emotional challenges experienced by women affected by endometriosis. Subcategories include (a) psychological distress, (b) worry about passing the disease on to female offspring, (c) worry about the future, (d) worry about the patient’s family member, (e) worry about not getting pregnant, (f) facing the intense stress of the illness, (g) lack of support from spouse and family member, (h) feelings of isolation in times of suffering and trying to fill the emotional gaps, (i) frustration with the behavior of others, (j) concealing the illness from others to avoid added stress, k) loss of hope for normalcy, l) feeling trapped in suffering until death, m) escaping painful memories at the peak of illness, n) continuous pain and suffering, o) positive or negativity challenges in dealing with others, p) fatigue from being ill, q) ineffectiveness of infertility treatments, frustration, and tensions caused by infertility.

##### Psychological distress

[I had no morale and felt no nerves, constantly arguing with everyone as if I owed them all. In my darkest moments, I couldn’t even recognize my husband, children, or mother. (Participant 4, from lower limit)]

##### Worry about passing the disease on to female offspring

[I am concerned about my daughter contracting the infection, what steps can I take to protect her? (Participant 5, from upper limit)]

##### Worry about the future

[My mind is consumed by sickness, fearing the possibility of cancer developing. (Participant 9, from lower limit)]

##### Worry about the patient’s family member

[The pain reflected in my child’s eyes, as my husband and child endured alongside me, their vitality surpassing my own. (Participant 9, from lower limit)]

##### Worry about not getting pregnant

[I felt like a trapped spring, my tears fueled by the fear of my husband and family’s reactions if I could not have children. (Participant 2, from upper limit)]

##### Facing the intense stress of the illness

[During ultrasounds and tests, stress always grips me. When the doctor mentioned tumor markers, my heart sank, and I teetered between life and death until the test results arrived. I anxiously awaited the outcome, my mood sour as I fixated on the doctor’s every word. (Participant 6, from upper limit)]

##### Lack of support from spouse and family member

[When my mother-in-law battled cancer, she garnered empathy and support. However, few understand the struggles of endometriosis. From sudden pains at social events to the inability to explain my absence, the ordeal remains unseen by others. Despite always empathizing, I now seek understanding in return as fatigue sets in. (Participant 10, from upper limit)]

##### Feelings of isolation in times of suffering and trying to fill the emotional gaps

[My husband, a devoted father, falls short as a partner, seemingly unaware of my needs. The resulting loneliness weighs heavily. The notion of staying together solely for the child is a daunting one. (Participant 3, from upper limit)]

##### Frustration with the behavior of others

[I felt upset when colleagues were sympathetic and prying about my illness. I needed support from my family—my father and sister didn’t sympathize, but they supported me, which made me feel better. Their encouragement was valuable. (Participant 3, from upper limit)]

##### Concealing the illness from others to avoid added stress

[Only my mother cried, and I requested everyone not to inquire about my illness. It was challenging for me to discuss it, even with my mother and sister, as I would insist that I was fine. (Participant 4, from lower limit)]

##### Loss of hope for normalcy

[They tell you that you won’t recover, and that feeling stays with you. It’s almost like wishing for death. (Participant 6, from upper limit).

##### Feeling trapped in suffering until death

[I don’t believe its cancer; you know what it is, but it’s worse than cancer. Sometimes I think about death, wondering if it could be a release. (Participant 10, from upper limit)]

##### Escaping painful memories at the peak of illness

[I try not to dwell on it too much. Thoughts of my illness make my heart ache, and I feel unwell. I prefer not to revisit those memories from that time. (Participant 4, from lower limit)]

##### Continuous pain and suffering

[My illness is always on my mind, pondering what the future holds. (Participant 6, from upper limit)]

##### Positive or negativity challenges in dealing with others

[My family must understand my menstrual cycle to comprehend my state. I become more sensitive during menstruation. Severe menstrual pain makes me irritable and weak, but my family shows patience. I later regret my actions and apologize. (Participant 8, from upper limit)]

##### Fatigue from being ill

[I have been exhausted by this illness for years. I sense failure and cannot persist in these circumstances. (Participant 10, from upper limit)]

##### Ineffectiveness of infertility treatments, frustration, and tensions caused by infertility

[I conceived effortlessly initially, and my child is now 6 years old. Despite desiring another pregnancy, I faced difficulties. I explained to my husband my struggles with conception, sought medical advice, and underwent treatment, only to discover I had endometriosis. (Participant 9, from lower limit)]

In general, the mental decline from endometriosis in affected women can be summarized as the heavy shadow it casts on their lives, imposing a significant psychological burden that may necessitate psychological interventions in some cases.

#### Social isolation

This concept illustrates the detrimental effects of endometriosis on women’s active participation in social activities in their personal and social lives. The subcategories include (a) endometriosis as a hindrance to career progression and (b) endometriosis as an obstacle to engaging in social activities.

##### Endometriosis as a hindrance to career progression

[These pains hindered my work as a hairdresser. My illness halted my progress, preventing me from achieving my goal of opening a dressing salon. When my health deteriorated and my husband discouraged me from working, attributing it to the poison of overwork, my life was completely disrupted by endometriosis. (Participant 4, from lower limit)]

##### Endometriosis as an obstacle to engaging in social activities

[Endometriosis caused me to miss work and studies. The pain was unbearable during my periods, forcing me to leave various work situation. I frequently had to skip work, citing the pain as the reason. (Participant 13, from lower limit)]

Overall, women with endometriosis often have to set aside their ambitions and aspirations, relinquishing their long-term goals.

#### Marital relationship disorder, and sexual dissatisfaction

This concept indicated the negative impact and sexual harassment of endometriosis in the married life of women suffering from this disease. The subcategories included (a) concealing the physical, psychological, and financial challenges of endometriosis from the spouse, (b) marital and sexual problems, and (c) feelings of inferiority and loss of femininity.

##### Concealing the physical, psychological, and financial challenges of endometriosis from the spouse

[Illness drains a person’s energy. I endure the pain silently, not wanting to upset my husband. Only a specific position allows us to intercourse. (Participant 10, from upper limit)]

##### Marital and sexual problems

[Intercourse is excruciating, but I hide my suffering from my husband to avoid him disapproval. (Participant 2, from upper limit)]

##### Feelings of inferiority and loss of femininity

[Why can’t I be like other women? The inability to conceive again and the constant pain and isolation haunt me. (Participant 9, from lower limit)]

In essence, women with endometriosis feel they have lost their femininity, attractiveness, and ability to satisfy their spouses. They view themselves as imperfect, struggling with pain, vaginal dryness, and bleeding during intercourse. This inner turmoil is a heavy burden for these women.

## Discussion

The main new concept derived from the qualitative data in the present study was “The Dominance of the Black Cloud of Endometriosis on Different Aspects of the Life of Women with Endometriosis” particularly highlighted through a subcategory termed “Physical depreciation caused by endometriosis, psychological deterioration caused by endometriosis, social isolation, marital relationship disorder, and sexual dissatisfaction. Prior research has demonstrated that women with endometriosis may experience a reduced quality of life, sexual problems, and marriage-related challenges [27, 28]. Complications like dyspareunia can have detrimental effects on intimacy and marital bonds [29, 30]. Qualitative data further highlights the adverse impact of endometriosis across various facets of women’s lives. Endometriosis consumes the lives of affected women similar to a dark shadow. The affected women expressed experiences of physical strain from endometriosis, mental difficulty, social seclusion, and disturbances in marital and sexual relationships. Their accounts highlighted challenges such as intense menstrual pain, heavy bleeding, cysts, gastrointestinal issues, continuous medication reliance, and struggles with daily life management. The qualitative study by Moradi et al. in Australia aimed to explore the impact of endometriosis on women’s lives and develop the EIQ tool using content analysis. The study identified key concepts including symptoms, delays in diagnosis and treatment, experiences with healthcare providers, and lack of information. In this research, women affected by the disease expressed discontent with its various effects. They highlighted issues such as menstrual pain, post-coital pain, heavy menstrual bleeding, medical negligence, and the lack of timely diagnosis and referral for severe symptoms. These challenges typically began during adolescence, with risk symptoms often being overlooked or normalized. Women spoke of the burden of relying on multiple hormonal medications, ineffective infertility treatments, and frustration with the healthcare system’s failure to promptly diagnose and refer adolescents showing risk symptoms, along with inadequate responses to the needs of those affected. Difficulty accessing endometriosis specialists, lack of communication from healthcare providers about the disease, along physical and mental health issues, strained relationships, and challenges to femininity were also noted. The lack of definitive treatments, and disruptions to daily life, including divorce due to infertility and difficulties in intimacy, were significant hardships. They discussed avoiding social engagements due to pain or bleeding, missing key life opportunities like marriage and education, and resorting to substance use to manage pain. Guidance on lifestyle modifications like proper diet, exercise, and cessation of smoking was lacking. Women shared their struggles with accepting and coping with the disease rather than tackling it head-on. They also revealed that maintaining relationships was particularly challenging, with the pain of intimacy leading partners to shy away from physical closeness [5]. Roomaney et al.‘s (2018) study was conducted in South Africa to elucidate the key aspects of women’s quality of life with endometriosis through a content analysis approach. The analysis revealed the following themes: Medical factors and diagnostic delay, obtaining information and knowledge, reducing stress related to the disease, receiving healthcare treatment, and doctor’s attention, especially concerning the financial aspects of patient care, dealing with financial effects such as high costs and lack of insurance support [31]. Namazi et al. (2019) conducted a qualitative study in Iran to explore the impact of endometriosis on affected women and develop the ERHQ tool through content analysis. Their findings revealed themes of “physical suffering, marital instability, mental disorders, social life disruption, and challenges in self-care.” Women reported various effects of endometriosis, including “menstrual irregularities, debilitating pelvic pain, emotional strife with partners, sexual dissatisfaction, feelings of despair and self-blame for infertility, isolation, disruptions in daily activities, lifestyle changes, and pain management difficulties “ [32]. Drabble et al. (2021) explored the complexities of pain from endometriosis in England, utilizing content analysis. Affected women described their pain as “dysmenorrhea, non-menstrual pelvic pain, dyspareunia, dyschezia, Urinary pain and burning, bladder-related pain, lower back and extremity pain (including groin and sciatica), upper body pain (chest, lung, upper back, shoulder), headaches (including migraines), ovulation pain, and kidney pain [33]. Riazi et al. (2014) conducted a study in Iran to examine the occurrence and diagnosis of endometriosis using a content analysis approach. A key theme identified was “feeling unable to fulfill the role of femininity,” which encompassed categories such as “dyspareunia and infertility.” Affected women reported intimate pain through various expressions, including complaints of pain during intercourse, relief after intercourse, intense pain during sex, sexual impotence, and sensations of burning during intimacy, as well as issues related to infertility, such as unsuccessful treatments and abortion histories [34]. In a similar vein, Jones et al. (2004) researched the impact of endometriosis on quality of life in England, employing a content analysis approach. This study found that endometriosis-related symptoms affect quality of life in a multifaceted way, extending beyond just psychosocial issues. Key themes identified include “unfavorable physical appearance, a sense of lack of control over daily life, feelings of powerlessness, social isolation, and concerns about their daughters’ health” [35]. The study by Hallstam et al. (2018) in Sweden explored the experience of living with painful endometriosis, emphasizing the struggle for coherence. The primary theme was “Living with painful endometriosis and the struggle for coherence,” supported by categories such as “ruined life (rules of endometriosis),” “dependency (help or harm),” and “the woman with painful endometriosis (‘I am different’).” These themes highlighted the feelings of being different, dependent, and burdened by a disrupted life, alongside dimensions of “questions, chaos, and meaningless suffering.” The experience of chronic and severe endometriosis reflects a struggle for coherence that healthcare providers must understand as a vital step in enhancing care for affected women [36].

Various research studies have demonstrated that endometriosis profoundly impacts the psychological well-being of affected women, imposing considerable mental counseling. Those with endometriosis commonly experience issues like anxiety, depression, and mood fluctuations, necessitating occasional psychological support [27, 28]. Many statements from the affected women in these studies align with the current research, but here, cases are more thoroughly classified into well-defined concepts, sub-classes, and sub-sub-classes. For instance, the concept of the “black cloud of endometriosis on the lives of women with endometriosis,” encompassing physical, psychological, social, and marital challenges faced by these women, has not been identified in previous studies. New concepts from this research include the intensification of pain post-cesarean surgery, gastrointestinal complications, concerns about the future and family, inadequate support from spouses and others, feelings of isolation during suffering, attempts to fill emotional voids, frustration with others’ treatment, concealment of the illness to reduce mental stress, a sense of perpetual suffering, avoidance of painful memories, and challenges—both positive and negative—in social interactions, along with the burden of financial issues related to endometriosis, all of which were not addressed in earlier studies.

Another new concept in the present study was “Psychological deterioration” derived from the qualitative data in this study. Conversations with women grappling with endometriosis revealed their experiences of psychological distress, concerns among mothers with the condition for their daughters, apprehensions regarding future implications of the disease, worries of family members, anxieties related to infertility, and the stress of managing the condition, the struggle to return to a sense of normalcy, the persistent feeling of being trapped in suffering until death, avoidance of revisiting painful memories associated with the peak of the illness, enduring ongoing pain, and challenges in interactions with others, fatigue stemming from the condition, as well as the despair and strain induced by infertility. In the treatment of women affected by this condition, addressing psychological issues and providing counseling are crucial. Even though only one woman in the current study sought psychological counseling to address symptoms and challenges stemming from endometriosis, all participants acknowledged the necessity for specialized psycho-emotional, sexual, and couples therapy for themselves and their partners. Women enduring endometriosis acknowledged the profound psychological counseling it had taken on them. However, these observations are solely based on the personal accounts of those affected, indicating a lack of psycho-emotional support or counseling for them and their partners within healthcare facilities, a critical need that remains unmet within the current system. Women with endometriosis expressed difficulties in marital relationships and lack of sexual satisfaction. They mentioned concealing physical, mental, and economic issues from their spouses, as well as marital and sexual challenges, feelings of inferiority, and loss of femininity. They reported problems like vaginal dryness and decreased libido due to hormonal medications, avoiding intimacy due to disease-related sexual problems, dissatisfaction with living conditions and marriage, and post-intercourse pain. Moreover, findings from the quantitative analysis in the present study revealed that women with endometriosis constantly worry about infertility, fear reactions from their husbands and others, and express concerns about not conceiving. They also experience a persistent fear of infertility and may consider egg freezing, particularly among young women. Past studies have indicated that endometriosis can adversely affect the education and training of women with the condition. The qualitative results of the present study also supported these findings, with women suffering from endometriosis expressing that the condition serves as a hindrance to both their work and education. They cited menstrual pain and various other associated issues as barriers to pursuing further education and being present in academic and professional settings. They vocalized that endometriosis impedes their professional progress, interferes with their aspirations and dreams, creates obstacles to working efficiently due to menstrual pain, and hampers their ability to fulfill job responsibilities and pursue ongoing education. The physical challenges, stress, and social limitations associated with endometriosis were highlighted as inhibiting factors in the workplace and in engaging in social activities.

### Strengths and limitations of the study

In this study, sampling was carried out in Mashhad. Participants were selected from the endometriosis clinic at Imam Reza Hospital, serving individuals from diverse urban backgrounds. All participants were confirmed to have endometriosis through histological diagnosis and approval from the clinic head. The study was supervised by an endometriosis expert for data validity and reliability. Participants were intentionally chosen for maximum diversity. A significant strength was the 98% high response rate. In this study, 14 women with endometriosis participated in the qualitative section, while 200 individuals were involved in the quantitative section. These women expressed a need for a medical professional who could provide information about their condition and allow them to share their concerns. They responded to 163 questions and willingly participated in the interviews for the qualitative portion. We extend our gratitude to all participants in this study. In the study’s second phase, data was collected through interviews. Women may withhold private information out of shame or embarrassment. To address this, interviews were conducted in a private setting between the researcher and participant, ensuring complete confidentiality without divulging names or details, and the process of receiving care remained unaffected. Since this study was conducted in the Mashhad metropolis, its findings may not be applicable to women with endometriosis in rural areas.

## Conclusion

Considering endometriosis as a chronic condition with no definitive treatment that significantly impacts women’s lives, it is essential to explore the needs of affected women for effective management and support. Tailoring services for women with endometriosis to address their specific requirements by understanding their challenges and obstacles throughout long-term treatment is crucial. This study aims to increase awareness among women with endometriosis, healthcare providers, and policymakers to identify their needs and recognize the barriers they face in various aspects of the lives of affected women with endometriosis. Therefore, implementing strategies to improve and optimize the challenges for women with endometriosis in healthcare systems is crucial, ultimately enhancing their quality of life with this chronic condition that lacks a cure. The results of this study indicate that endometriosis harms various aspects of women’s lives, including physical health, psychological well-being, social interactions, sexual and marital relationships, economic factors, employment and occupational dimensions, education, and lifestyle. As a result, the healthcare system, management, and policy-making, socioeconomic status, lack of awareness, culture, as well as the attitudes of women and healthcare providers, are all important in the treatment of women with endometriosis. These factors can guide future research.

## Electronic Supplementary Material

Below is the link to the electronic supplementary material.

Supplementary Material 1

## Data Availability

The original contributions presented in the study are included in the article. Further inquiries can be directed to the corresponding author.

## Footnotes

1 Consolidated criteria for reporting qualitative studies (COREQ): 32-item checklist.
