# Supplementary material for: The Black Cloud of Endometriosis on different aspects of the Life of Women with Endometriosis: a qualitative study in Iran
Source: BMC Psychiatry. 2024 Dec 27;24:957. doi: 10.1186/s12888-024-06405-8 (PMC11673853; doi:10.1186/s12888-024-06405-8)
Supplement: Supplementary file 1 — Supplementary Material 1 [file 12888_2024_6405_MOESM1_ESM.doc]

**Qualitative Interview Guide**

1. How did you first learn about endometriosis and what was your initial reaction?
2. Please provide a brief overview of your daily routine.
3. Can you share your perspective and personal experiences related to your illness?
4. How has your life been impacted since being diagnosed with this disease?

To gather detailed information, probing questions like "explain further" and "clarify your point" were used. Concluding the interviews, open-ended questions such as "Is there anything else you'd like to discuss?" were asked. The final query was about any missed topics the interviewee finds important. Interview lengths varied from 60 to 120 minutes based on participant availability.
